# Fine Mapping of the Interaction between C4b-Binding Protein and Outer Membrane Proteins LigA and LigB of Pathogenic *Leptospira interrogans*

**DOI:** 10.1371/journal.pntd.0004192

**Published:** 2015-10-30

**Authors:** Leandro C. D. Breda, Ching-Lin Hsieh, Mónica M. Castiblanco Valencia, Ludmila B. da Silva, Angela S. Barbosa, Anna M. Blom, Chang Yung-Fu, Lourdes Isaac

**Affiliations:** 1 Department of Immunology, Institute of Biomedical Sciences, University of São Paulo, São Paulo, Brazil; 2 Department of Population Medicine and Diagnostic Sciences, College of Veterinary Medicine, Cornell University, Ithaca, New York, United States of America; 3 Laboratory of Bacteriology, Institute Butantan, São Paulo, Brazil; 4 Department of Translational Medicine, Division of Medical Protein Chemistry, Lund University, Malmo, Sweden; Medical College of Wisconsin, UNITED STATES

## Abstract

The complement system consists of more than 40 proteins that participate in the inflammatory response and in pathogen killing. Complement inhibitors are necessary to avoid the excessive consumption and activation of this system on host cells. Leptospirosis is a worldwide zoonosis caused by spirochetes from the genus *Leptospira*. Pathogenic leptospires are able to escape from complement activation by binding to host complement inhibitors Factor H [FH] and C4b-binding protein (C4BP) while non-pathogenic leptospires are rapidly killed in the presence of fresh serum. In this study, we demonstrate that complement control protein domains (CCP) 7 and 8 of C4BP α-chain interact with the outer membrane proteins LcpA, LigA and LigB from the pathogenic leptospire *L*. *interrogans*. The interaction between C4BP and LcpA, LigA and LigB is sensitive to ionic strength and inhibited by heparin. We fine mapped the LigA and LigB domains involved in its binding to C4BP and heparin and found that both interactions are mediated through the bacterial immunoglobulin-like (Big) domains 7 and 8 (LigA7-8 and LigB7-8) of both LigA and LigB and also through LigB9-10. Therefore, C4BP and heparin may share the same binding sites on Lig proteins.

## Author Summary

Leptospirosis is a neglected infectious disease of public health significance. It is one of the most important zoonosis worldwide, affecting at least 500,000 people each year of which approximately 10–20% of cases are fatal. Most of the cases occur in tropical developing countries which lack proper sanitation facilities. The complement system plays an important role to control this infection and pathogenic leptospires developed several strategies to escape from this system, for example, binding to complement regulatory proteins such as C4b binding protein (C4BP). In this work, we fine mapped the C4BP domains important for this evasion. We also mapped C4BP interactions with LigA and LigB, two surface proteins present in all pathogenic leptospires. The available leptospirosis vaccines are based on region-specific serovars and are not very protective. Consequently, the search of better vaccines is necessary and Lig A and Lig B represent promising vaccine candidates to immunize people worldwide. We believe that deeply understanding the interactions between C4BP with LigA and LigB could open new perspectives for immunotherapy and development of new drugs to be used during the first days of infection, when the production of specific antibodies is still low and the complement system plays an important role to control this infection.

## Introduction

Leptospirosis is a worldwide zoonosis caused by spirochetes from the genus *Leptospira* [1]. Rodents are the main reservoir of *Leptospira*, which can be excreted in animal urine [2]. The disease is transmitted by contact of abraded skin or mucosal membranes with water contaminated with infected urine and it presents a higher incidence in developing countries, which lack proper sanitation conditions. In developed countries, leptospirosis is more frequently associated with agriculture and aquatic sports. According to the World Health Organization [WHO], more than 500,000 cases of leptospirosis are diagnosed per year worldwide and the mortality rates reach 10% in some areas. Since 80–90% of the patients initially present nonspecific symptoms, such as headache, fever and joints pain that may disappear without any specific treatment, these epidemiological data may be underestimated [3–4]. So far, 13 pathogenic and 6 saprophytic species with more than 320 serovars (260 pathogenic and 60 saprophytic) of *Leptospira* species have been described [5]. Recent studies on the differences between pathogenic and saprophytic *Leptospira* have contributed to our knowledge concerning the mechanisms that allow survival of pathogenic strains inside the host. In 2004, Koizume and Watanabe described two surface proteins expressed only in pathogenic *Leptospira*: Leptospiral immunoglobulin-like A (LigA) and B (LigB)[6–8]. During the last decade several groups have been studying the functions of Lig proteins and their contributions to *Leptospira* infection. A well-established role for LigA and LigB is their capacity to interact with multiple extracellular matrix components such as fibrinogen, fibronectin [9] collagen [10] laminin and elastin [11]. Saprophytic *Leptospira* expressing recombinant LigA or LigB proteins present stronger adhesion to eukaryotic cells and to fibronectin in vitro [12]. A fragment of the LigA protein has been shown to be a promising vaccine candidate, conferring high-levels of protection in hamster models of leptospirosis [13–14].

Lig proteins have also been shown to contribute to pathogenic *Leptospira* immune evasion by binding to the complement system inhibitors Factor H (FH), FH-like 1 (FHL-1), FH-related 1 (FHR-1) and C4b-binding protein (C4BP)[15]. In addition, LcpA, another surface protein present exclusively in pathogenic *Leptospira*, binds to C4BP [16], FH and vitronectin [17]. The acquisition of such inhibitors on the bacterial surface potentially enables pathogenic *Leptospira* to down-regulate all pathways of this system. FH is a 150 kDa protein composed of 20 control complement protein (CCP) domains (also known as short consensus repeat (SCRs)[18–19]. CCPs 1–3 interact with C3b which is important for FH’s role as a cofactor in Factor I (FI)-mediated cleavage of C3b [19]. FH cofactor activity is maintained when bound to Lig proteins [15]. FH also inhibits the interaction of Factor B with C3b, accelerating decay of the C3 convertase of the alternative pathway [20]. FH binds to LcpA mainly by CCP 20 [17] and to Lig proteins through CCPs 5 and 20 [15].

**Fig 1:**
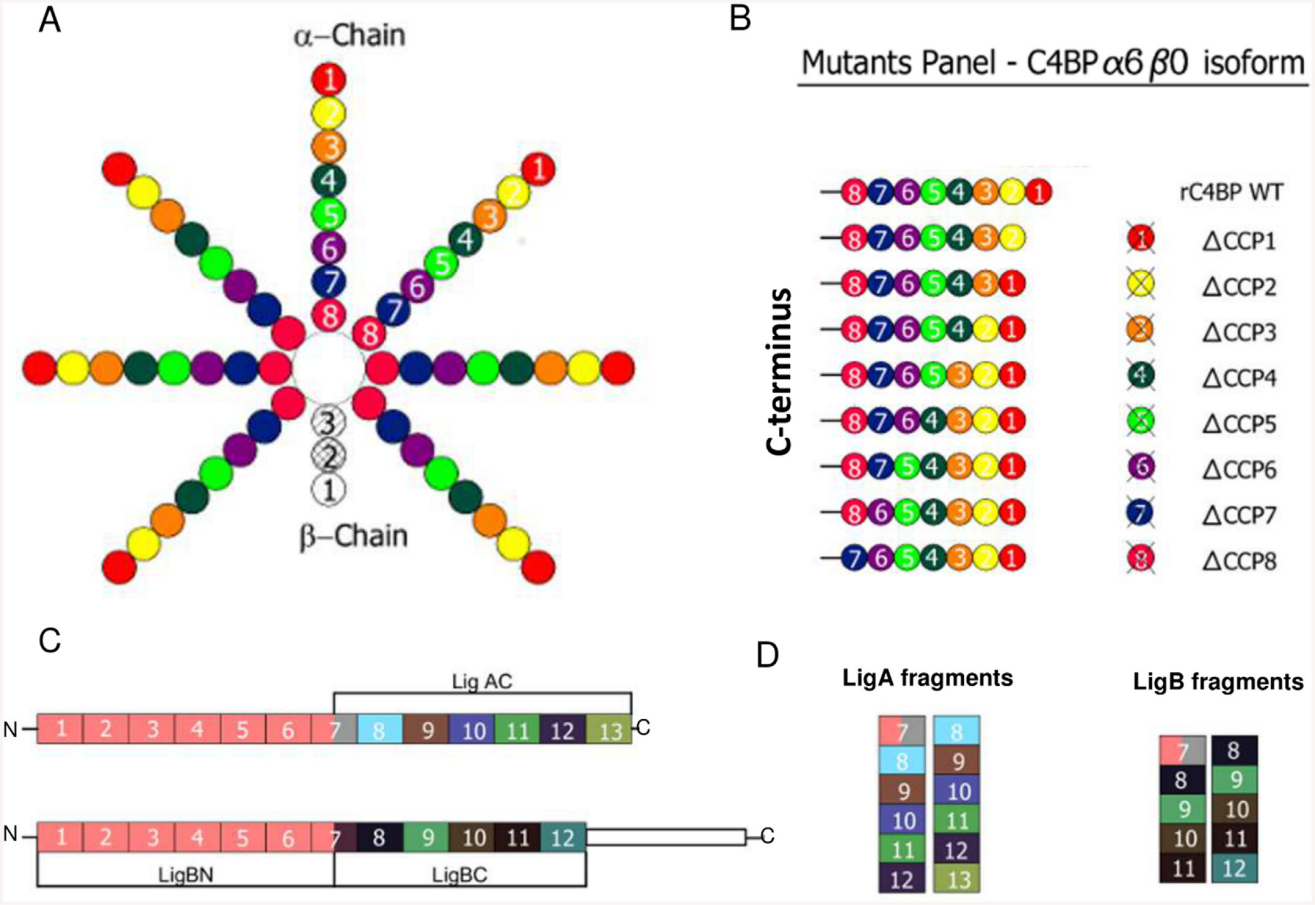
Schematic diagrams of C4BP molecule, C4BP recombinant mutants and *L*. *interrogans* proteins LigA and LigB. (**A**) Structure of human C4BP isoform α7β1 [4]. Each α-chain is composed of 8 complement control protein (CCP) domains while the β-chain is composed of 3 CCPs. CCP1 from the α and β-chains are localized at the N-terminus region and α-chain CCP8 and β-chain CCP3 are situated near the central core (C-terminus). (**B**) C4BP recombinant wild type and mutants (α6β0) used in this work. Each mutant is composed of 6 α-chains. Each wild type α-chains contains 8 CCPs while mutant α-chains are formed by only 7 CCP domains (Δ denotes which CCP is missing in each mutant). (**C**) Illustration of recombinant leptospiral immunoglobulin-like proteins (Lig)A (LigA) and B (LigB). LigA is composed of 13 bacterial immunoglobulin-like (Big) domain repeats while LigB is composed of 12 Big domains. The fragment corresponding to the first six and a half domains of LigA and LigB (residues 26–630; identical in both proteins) is named LigBN. The fragments that corresponding to the second half of Big domain 7 to the Big domain 13 of LigA (residues 631–1225), is named LigAC and fragments corresponding to the half of Big domain 7 to Big domain 12 of LigB (residues 631–1156), is named LigBC. (D) Schematic representation of the recombinant LigA and LigB fragments containing tandem pairs of Big domains.

C4BP is a 570-kDa glycoprotein and relatively abundant in plasma (200 μg/ml–500 μg/ml) [21]. The C4BP molecule is comprised of two different polypeptide chains: C4BP α chain (75 kDa) and C4BP β chain (45 kDa). In serum, three C4BP isoforms can be observed which differ in the stoichiometries of α and β chains: α7β1 (most common), α6β1 and α7β0 [22]. C4BP α chain contains eight CCPs and C4BP β chain contains three CCPs (Fig 1). C4BP inhibits the classical and the lectin pathways acting as a cofactor for the cleavage of C4b by FI. It also prevents binding of C2a to C4b and accelerates the decay of the C3 convertase (C4bC2a) of both pathways [23–25]. Binding sites for several ligands of C4BP have been localized using C4BP mutants. The alpha-chains CCP2 and CCP3 are crucial for the interaction with C4b [26–27] while binding to heparin requires CCPs 1–3 of the alpha chain [28]. The first three CCP domains of the alpha chain are also involved in interactions with several bacterial pathogens. C4BP also interacts with protein S through its beta-chain CCP1 [29–31]. In a previous study, we showed that LigA and LigB interact with C4BP in a dose-dependent manner and that bound C4BP remains functionally active, mediating degradation of C4b by FI [15]. In this study, we focused more closely on the interaction of Lig proteins with C4BP. Using a panel of C4BP mutants, we mapped the CCPs involved in the interaction with whole *Leptospira* and specific LigA and LigB domains. We show that ionic forces play a role in the binding of C4BP to Lig proteins and that the interaction is inhibited by heparin, a known C4BP ligand.

## Materials and Methods

### Ethics statement

All the experiments involving laboratory animals were evaluated by the “Ethics Committee for Animal Use” from Institute of Biomedical Sciences—University of São Paulo (our Institutional Animal Care and Use Committee) and approved under the protocol number06/10/CEUA/ICB and 99/2/CEUA/ICB. The procedures are according to the Brazilian National Law number 11794 from 10/08/2008, which regulates all research activities involving animal use in the country.

### Bacteria and culture conditions

*L*. *interrogans* serovar Kennewicki strain Fromm was cultured in EMJH medium [Difco] supplemented with 10% of bovine serum albumin (BSA) during 7 days at 29°C under aerobic conditions. The virulence of this strain was confirmed in vivo upon infection in hamsters at the Laboratory of Bacterial Zoonosis of the Faculty of Veterinary Medicine and Zootechny, University of São Paulo.

### Proteins and antibodies

Human purified C4BP (α7β1) was purchased from Complement Technology (Texas, USA). Recombinant wild type C4BP (rC4BP) and C4BP mutant α chains lacking single CCP domains α6β0 (Fig 1) were expressed in eukaryotic cells and purified by affinity chromatography as previously described [26, 32]. To exclude the possibility that polyclonal rabbit anti-human C4BP (Calbiochem) used in this study would interact preferentially with the C-terminal rather than to the N-terminal region of C4BP, which could potentially interfere with the detection of C4BP lacking the CCPs localized in this region, an ELISA was performed to guarantee that the antibody could recognize equally all C4BP mutants. All mutants were specifically recognized by anti-human C4BP. LigA is composed of 13 bacterial immunoglobulin-like [Big] domain repeats while LigB consists of only 12 Big domains. The first six and a half domains of LigA and LigB are identical (residues 26–630). This recombinant fragment was expressed and named LigBN. Fragments corresponding to the C-terminal half of the 7^th^ Big domain until 13th Big domains of LigA (residues between 631–1225) and LigB (residues between 631–1156) were named LigAC and LigBC. Cloning, expression, and purification of LigAC, LigBC, LcpA and LIC10301 (negative control) were described previously [15,16]. To pinpoint the C4BP binding sites on Lig proteins, a series of tandem Ig-like domains (also known as Big domains) of Lig proteins were expressed and purified as previously described [11]: LigA7-8, LigA8-9, LigA9-10, LigA10-11, LigA11-12, LigA12-13, LigA8-13 (or LigAC) (numbers correspond to Big domains of LigAC), or LigB7-8, LigB8-9, LigB9-10, LigB10-11, LigB11-12, LigB7-12 (or LigBC)(numbers correspond to Big domains of LigBC). Polyclonal mouse anti-LigA and anti-LigB antibodies were obtained in our laboratory after immunizing mice with 10 μg/each of purified recombinant proteins, using Al(OH)_3_ as adjuvant. Two booster injections of the same protein preparation were given at 2-week intervals. One week after each immunization, animals were bled from the retro-orbital plexus and sera pooled. The titers of anti-LigA and anti-LigB were determined by enzyme-linked immunosorbent assay (ELISA). Secondary antibodies conjugated with peroxidase or FITC were purchased from KPL (Maryland, USA) and Abcam (Cambridge, UK), respectively.

### Mapping CCPs α-chain of C4BP that interact with LcpA, LigAC and LigBC proteins and with whole *L*. *interrogans*

To map the regions of C4BP CCPα-chain which interact with LigAC, LigBC and LcpA, microtiter plates (Costar 3590) were coated with 1 μg of LigAC, LigBC, LcpA or BSA (negative control) overnight at 4°C. The wells were washed with PBS (137 mM NaCl, 2.7 mM KCl, 10 mM Na_2_HPO_4_, 1.8 mM KH_2_PO_4_), blocked with PBS containing 3% BSA (PBS-BSA) for 2 h at 37°C, and incubated with 1 μg of each recombinant C4BP fragment (diluted in PBS) for 60 min at 37°C. After three washes with PBS containing 0.05% Tween 20 (PBS-T), C4BP bound to *L*. *interrogans* recombinant proteins was detected with rabbit polyclonal anti-human C4BP (1:2000 in PBS-T containing 1% BSA), followed by peroxidase-conjugated anti-rabbit IgG (1:5000 in PBS-T 1% BSA). Substrate reaction was performed with o-phenylenediamine dihydrochloride (Pierce), and absorbance was measured at 492 nm. The interaction between *L*. *interrogans* recombinant proteins with recombinant wild-type C4BP (rC4BP WT) was considered 100% binding. To map the CCP alpha-chains of C4BP that interact with intact *L*. *interrogans*, bacteria were harvested at 10.000 x g for 10 min, washed 2 times with PBS and suspended in 1 ml PBS. Suspensions were adjusted to 1x10^8^ leptospires using a Petroff-Hausser chamber under dark-field microscopy (Nikon Eclipse 50). Suspensions of *L*. *interrogans* were incubated with 2 μg of each C4BP mutant or with PBS for 2 h at room temperature. After washing, bacteria were incubated with polyclonal rabbit anti-human C4BP (1:150 in PBS/1% BSA; final volume: 40 μl), followed by FITC-conjugated anti-rabbit IgG (AbCam; 1:200 in PBS/1% BSA). Geometric mean fluorescence intensity (GMFI) was measured and the binding of *L*. *interrogans* to rC4BP WT was set up as 100%. The auto fluorescence of *Leptospira* was discounted before plotting the data. It is worth to mention that Matsunaga and colleagues (2005) showed that the expression of LigA and LigB proteins by *L*. *interrogans* is determined by environmental signals [33]. Thus, to perform this experiment we used low-passage forms of *L*. *interrogans* obtained from hamster. Before we used we confirmed the expression of LigA and LigB proteins on *L*. *interrogans* by Western blot and flow cytometry.

### Mapping LigAC and LigBC regions that interact with C4BP

Microtiter plates were coated with 1 μg of each LigA or LigB fragment overnight at 4°C in PBS (130 mM NaCl, 7 mM Na_2_HPO_4_, 3 mM NaH_2_PO_4_). After blocking with 3% BSA, C4BP (1μM—0.0156 μM, 2 fold serial dilution) was then added to the plates for 1 h at 37°C. Between each step, plates were washed with PBS-T three times. Subsequently, mouse monoclonal anti-C4BP antibodies (1:2000) (EMD Millipore) recognizing C4BP α- and β-chains were used as primary antibodies. Horseradish peroxidase (HRP)-conjugated rabbit anti-mouse IgG antibody (1:2000) (Invitrogen) was used as secondary antibody. After washing three times with PBS-T, 100 μl of 0.2 mg/ml 3,3’,5,5’-tetramethylbenzidine (TMB) substrate (Kirkegaard & Perry Laboratories) was added to each well. Finally, after a 10 min-incubation the microtiter plates were read at 630 nm using an ELISA plate reader (Biotek EL-312). Each value represents the mean ± SE of three independent experiments, each performed in triplicate.

### Assessment of the binding of LigAC and LigBC to heparin and competition assay

To confirm the importance of the C4BP C-terminal region on the interaction with LigAC and LigBC proteins, we performed a competition assay with heparin, which interacts with C4BP CCP 1, CCP 2 and CCP 3 [28]. ELISA was performed as described above. Briefly, plates were coated with LigAC or LigBC proteins. After blocking and washing 1 μg of C4BP was mixed with different amounts of heparin and added to the reaction. C4BP bound to LigAC or LigBC proteins was detected using anti-C4BP. To verify if Lig proteins interact directly with heparin, microtiter plate wells were coated with 1 μg of heparin overnight. After washing and blocking, 1μg of LigAC or LigBC was added to the reaction. After three washes, LigAC and LigBC bound to heparin were detected using mouse anti-LigA or mouse anti-LigB, respectively, (1:1000 in PBS-T containing 1% BSA) followed by peroxidase-conjugated anti-mouse IgG (1:5000 in PBS-T 1% BSA).

To identify which Big domains of LigA and LigB proteins interact with heparin, heparin-BSA conjugates were prepared as previously described [34]. Free BSA was separated from the heparin-BSA by Superdex-75 size exclusion column (GE Healthcare). Plates were coated with 1 μg of heparin-BSA or BSA (negative control) at 4°C overnight. After blocking with PBS-BSA for 2h, different concentrations of Lig protein constructs were applied to heparin-coated wells for 1h at 37°C. Between each step, the wells were washed with PBS-T three times. To evaluate the heparin binding ability of each Lig construct, mouse monoclonal antibodies (1:1000) against Lig proteins were used as primary antibodies and HRP-conjugated rabbit anti-mouse IgG antibody (1:2000) (Invitrogen) was used as secondary antibody with TMB as substrate. In order to examine if the C4BP binding sites on Lig proteins are located near the heparin binding regions, a competitive ELISA was performed. Firstly, Lig protein constructs, which can potentially interact with C4BP, were immobilized overnight on microtiter plates at 4°C. After blocking with PBS-BSA for 2h, 1 μg of C4BP mixed with different concentrations of heparin (0–10 mg/ml) were added to the plates. Between each step, the microtiter wells were washed with 0.05% PBS-T for three times. Subsequently, mouse monoclonal anti-C4BP α/β chains antibodies (1:2000) (EMD Millipore) and HRP-conjugated rabbit anti-mouse IgG antibodies (1:2000) (Invitrogen) were used respectively as primary and secondary antibodies to detect the binding levels of C4BP to different Lig protein fragments. C4BP binding to each Lig protein fragment in the absence of heparin was set as 100%.

To estimate binding affinities for the interaction between LigAC or LigBC with C4BP or with heparin, increasing concentrations of Lig proteins were incubated with a fixed amount of C4BP (1μg) or heparin (1μg). Using GraphPad Prism 5.0 (GraphPad Software, Inc.), the dissociation constant (K_d_) was estimated by nonlinear regression, using the equation Y = Bmax*X/(K_d_ + X).

To test if the interaction of C4BP with LigA, LigB and LcpA is dependent on ionic strength an ELISA assays [as described above] were performed in the presence of increasing amounts NaCl [0 to 600 mM] diluted in 10 mM Na_2_HPO_4_ and 1.8 mM KH_2_PO_4_.

### Statistical analysis

GraphPad Prism 5.0 (GraphPad Software, Inc.) was used for statistical analyses and ANOVA were used to analyze the data.

## Results

### Mapping the binding sites of LcpA, LigAC, LigBC and whole *L*. *interrogans* on C4BP protein

**Fig 2:**
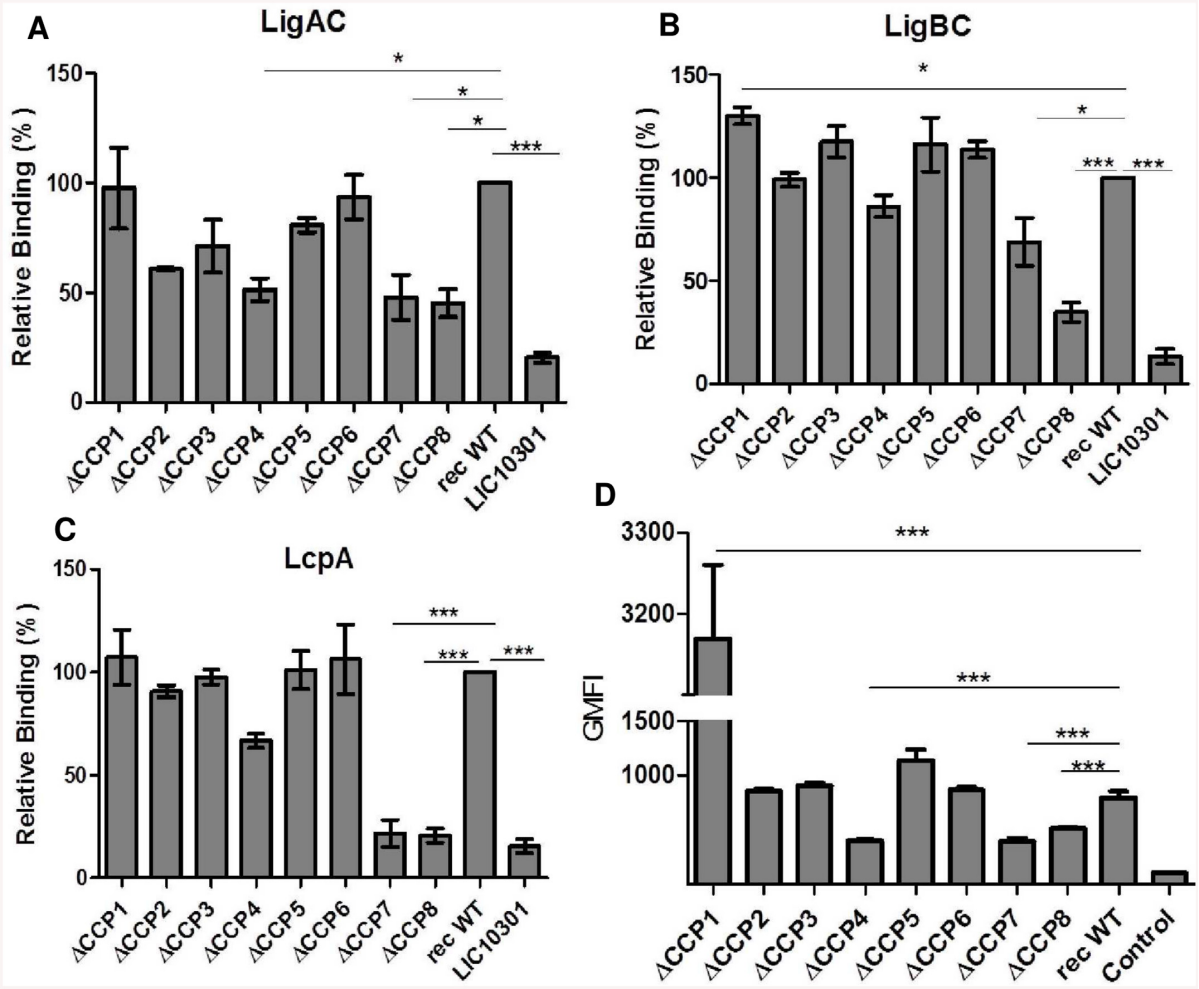
Binding of recombinant proteins LcpA, LigAC, LigBC and whole *L*. *interrogans* to recombinant mutant C4BP molecules. Microtiter plates were coated with LigAC (**A**), LigBC (**B**), LcpA (**C**) or LIC10301 (as negative control). After adding of each C4BP recombinant mutant or wild type (rec C4BP WT) protein (described in Fig 1B), binding was measured using rabbit polyclonal anti-human C4BP and peroxidase-conjugated anti-rabbit IgG. Each point represents the mean absorbance value at 492 nm +/- the SD of 3 independent experiments each performed in triplicate. The interaction of recombinant proteins of *L*. *interrogans* with rec C4BP WT was set as 100% binding. (**D**) Binding of C4BP mutant proteins to whole *L*. *interrogans*. Leptospires (1x10^8^) were incubated with rec C4BP WT, C4BP mutants or PBS (negative control). To detect the C4BP binding to leptospires, polyclonal mouse anti-C4BP and FITC-conjugated anti-rabbit IgG were used. Each point represents the geometric mean fluorescence intensity (GMFI) +/- SE of 3 independent experiments each performed in triplicate. Data were analyzed using ANOVA test; *p<0.05; ***p<0.0001.

We used recombinant fragments of the entire C-terminus regions of LigA and LigB to map their binding to specific C4BP CCPs. LcpA, a confirmed leptospiral ligand for C4BP [16], was included in this study as a positive control. Using the panel of C4BP mutants lacking single CCPs in the α-chain (Fig 1A and 1B), we observed that interaction with LigAC (Fig 2A), LigBC (Fig 2B) and LcpA (Fig 2C) was significantly reduced in the absence of CCP7 and CCP8. In addition, we observed that CCP4 of C4BP is also important for the interaction with LigAC (Fig 2A). The C4BP ΔCCP4 mutant also showed a tendency towards reduced binding to LigBC and LcpA, though this reduction was not statistically significant (Fig 2B and 2C). C4BP CCP4, CCP7 and CCP8 domains were also found to be important for the binding of this regulatory protein directly to whole *L*. *interrogans* cells. Interestingly, the binding of *L*. *interrogans* to the C4BP mutant lacking the CCP1 domain of the α-chain was 3 times greater than that observed for C4BP WT (Fig 2D).

### Mapping the C4BP binding sites on LigA and LigB

**Fig 3:**
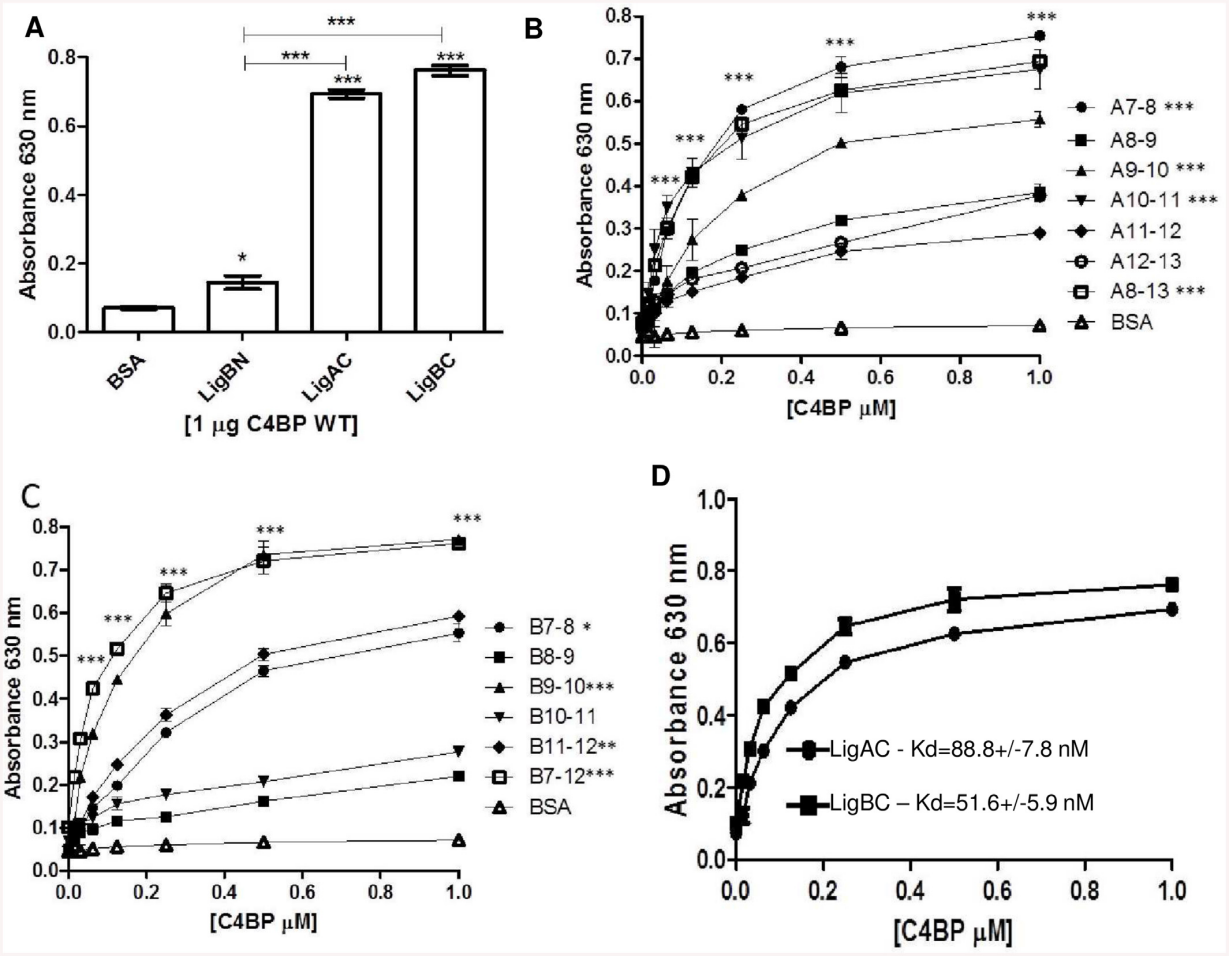
LigA and LigB Big domains are important for binding to C4BP. (**A**) The interaction of C4BP with the conserved N-terminus region of LigA and LigB proteins (LigBN), the C-terminus region of LigA (LigAC) and the C-terminus region of LigB (LigBC) was measured (**B-C**) The binding of two-domain serial constructs of LigAC (**B**) or LigBC (**C**) (described in Fig 1D) to C4BP was studied using increasing concentrations of C4BP. BSA was used as negative control. Each value represents the mean ± SD of three independent experiments, each performed in triplicate and binding of C4BP to the recombinant Lig proteins was compared with the binding of these molecules to BSA by the ANOVA (*p<0.05; **p<0.01 ***p<0.001) (**D**) Estimation of the dissociation constant (K_d_) for the interactions of LigAC and LigBC with C4BP. K_d_ was calculated by fitting the data to the equation Y = Bmax*X/(K_d_ + X) using GraphPad Prism 5.0 (GraphPad Software, Inc.). Each data point represents the mean +/- SD of three trials in triplicate. C4BP binding to leptospiral proteins was detected using rabbit anti-C4BP as primary antibody and anti-rabbit IgG conjugated with peroxidase as secondary antibody.

To investigate the C4BP binding sites on Lig proteins, wild type C4BP was added to microtiter plates coated with recombinant LigA and LigB truncations. Interaction of the variable C-terminus halves of both LigA (LigAC) and LigB (LigBC) with C4BP is significantly greater than that observed for the N-terminus region (LigBN, which is identical in both proteins) (Fig 3A). To further pinpoint the minimal C4BP binding sites on the two Lig proteins, a series of two-domain tandem Ig-like repeats (Big domains) across the variable C-terminus region of LigA and LigB were generated. The results indicate that all the LigA and LigB tandem repeats bind to C4BP but with different affinities. The major binding sites are located in fragments LigA7-8, LigA9-10, LigA10-11 (Fig 3B) and in fragments LigB7-8, LigB9-10 and LigB11-12 (Fig 3C). A minimal two-domain Ig-like repeat seems to be critical for the C4BP/Lig interaction since binding is significantly reduced when constructs containing only a single-Ig-like domain were used. We also observed that LigAC and LigBC affinities for C4BP are, respectively, K_d_ = 88.8 +/- 7.8 nM and K_d_ = 51.6 = /- 5.9 nM, (Fig 3D). From these results, we conclude that the variable regions of Lig proteins are the most important binding sites for C4BP, that the minimal binding motifs seem to be made up of pairs of Big domains and that the Big domains pairs displayed the strongest affinity for C4BP are domains 7–8, 9–10, 10–11 in LigA and domains7-8, 9–10 and 11–12 in LigB.

### Heparin inhibits the binding of LigAC and LigBC to C4BP

**Fig 4:**
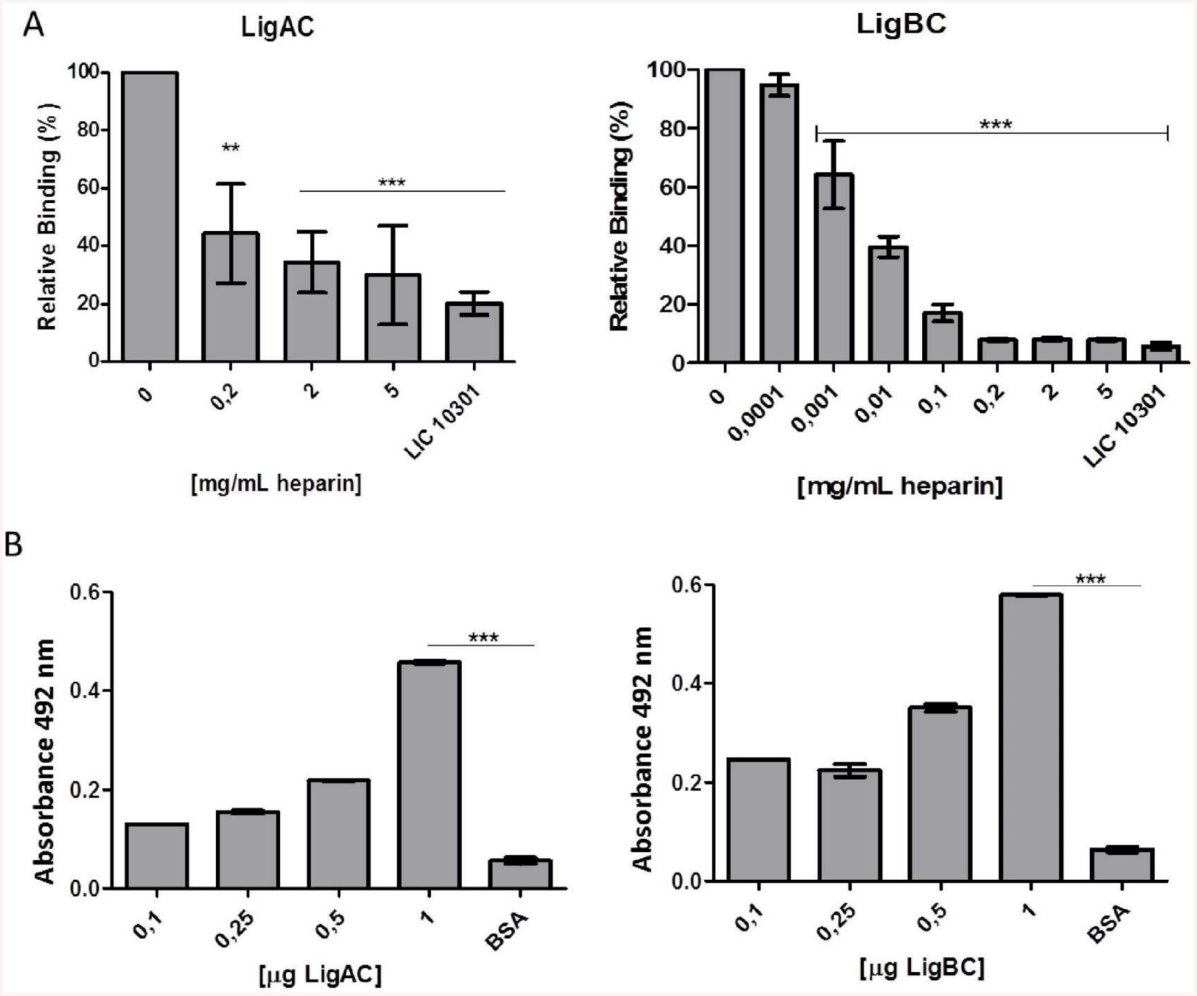
Interaction between C4BP and leptospiral recombinant proteins LigAC and LigBC is heparin dependent. (**A**) C4BP was incubated with different concentrations of heparin before adding to microtiter plates previously coated with LigAC or LigBC. Recombinant LIC 10301 was included as negative control. The binding of C4BP to the leptospiral proteins was detected using polyclonal rabbit anti-C4BP as primary antibody and anti-rabbit IgG conjugated with peroxidase as secondary antibody. Each point represents the mean absorbance value at 492 nm of 3 independent experiments +/- the SD, each performed in triplicate. The binding of recombinant proteins of *L*. *interrogans* to C4BP in the absence of heparin was considered 100% (** *p*<0.001; *** *p*<0.0001). (**B**) Microtiter plates were coated with heparin and then, different amounts of LigAC or LigBC (0.1 μg–1 μg) were added to the plates. Anti-LigA or Anti-LigB antibodies were used to detect the protein bound to immobilize heparin. BSA was used as negative control. Each point represents the mean absorbance value at 492 nm +/- the SD of 3 independent experiments each, performed in triplicate (*** *p*<0.0001).

The results in Fig 2 suggest the C4BP N-terminus domains CCP1, CCP2 and CCP3 do not contribute to binding with LigA and LigB. To further test this hypothesis, we investigated this interaction in the presence of the anionic polysaccharide heparin. Heparin is a component of the extracellular matrix and it is known to interact with C4BP CCP1, CCP2 and CCP3 of the α-chain [28]. Recombinant *L*. *interrogans* proteins were incubated with recombinant C4BP WT in the presence of different concentrations of heparin. Surprisingly, we observed that heparin inhibited the interaction of C4BP with LigBC even at concentrations as low as 10^-3^ mg/ml while the interaction of C4BP with LigAC was inhibited with 0.2 mg/ml of heparin (Fig 4A).

### LigA and LigB interact with heparin

**Fig 5:**
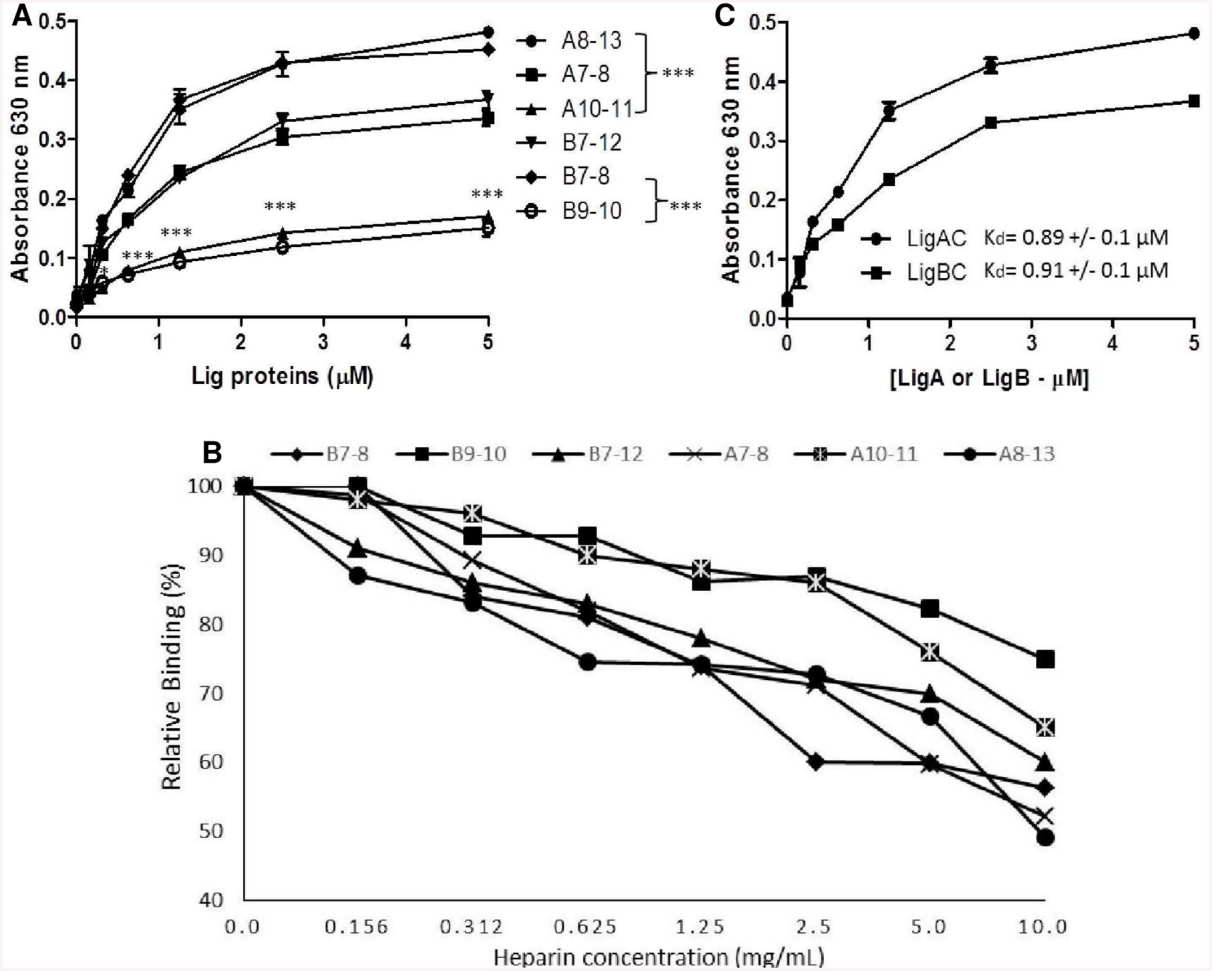
LigAC and LigBC interact with heparin mainly via Big 7 and Big 8. (**A**) Using different LigA and LigB tandem domain constructs, we observed that Big7-8 are the major heparin binding sites on the C-terminus variable regions of LigB and LigA. (**B**) Binding of C4BP to different LigA and LigB tandem domain constructs is affected by heparin. Immobilized Lig protein constructs were incubated with C4BP mixed with different concentrations of heparin. C4BP bound to each Lig protein fragments in the absence of heparin was set as 100%. Each point represents the mean absorbance value at 492 nm or 630 nm +/- the SD of 3 independent experiments each performed in triplicate. Binding of heparin to LigA7-8, LigA8-13 and LigB 7–8, LigB7-12 were compared to LigA10-11 and LigB 9–10, respectively. ***p<0,001 (**C**) Estimation of the dissociation constant (K_d_) for the interactions of LigAC and LigBC with heparin. K_d_ was calculated by fitting the data to the equation Y = Bmax*X/(K_d_ + X) using GraphPad Prism 5.0 (GraphPad Software, Inc.). Each data point represents the mean +/- SD of three trials in triplicate.

We then investigated if the above phenomenon could be a result of heparin binding directly to LigA and LigB. According to Ching and colleagues [35], recombinant LigBC binds to heparin, and this interaction may help *L*. *interrogans* to invade host cells. In the present study, we confirmed these results and additionally observed that LigAC interacts with immobilized heparin as well (Fig 4B). In order to verify if heparin and C4BP would compete for the same binding sites on LigA and LigB, we performed heparin binding experiments using recombinant fragments corresponding to pairs of Big domains from the C-terminus halves of LigA and LigB. Fig 5A shows that Big domain pairs 7–8 of both LigA and LigB are the most important regions for the interaction with heparin.

To test whether heparin and C4BP share binding sites on LigA and LigB proteins, a competition assay was performed using different LigA and LigB fragments. In the presence of the highest concentration of heparin used (10 mg/ml), the binding of C4BP to LigA7-8 and LigB7-8 was reduced by 50% and 45%, respectively. The interactions of C4BP with LigA10-11 and LigB9-10 were decreased by 35% and 27% (respectively) under the same conditions (Fig 5B). We also observed that LigAC and LigBC seem to have similar affinities for heparin (K_d_ = 0.89 +/-0.1 μM and K_d_ = 0.91 +/- 0.1 μM, respectively) (Fig 5C). Together, the above results suggest that there is significant overlap of the heparin and C4BP binding sites on LigA and LigB and that these shared binding regions include Big domains 7 and 8 from both leptospiral proteins.

### Interaction of C4BP with LigAC and LigBC proteins is ionic strength dependent

**Fig 6:**
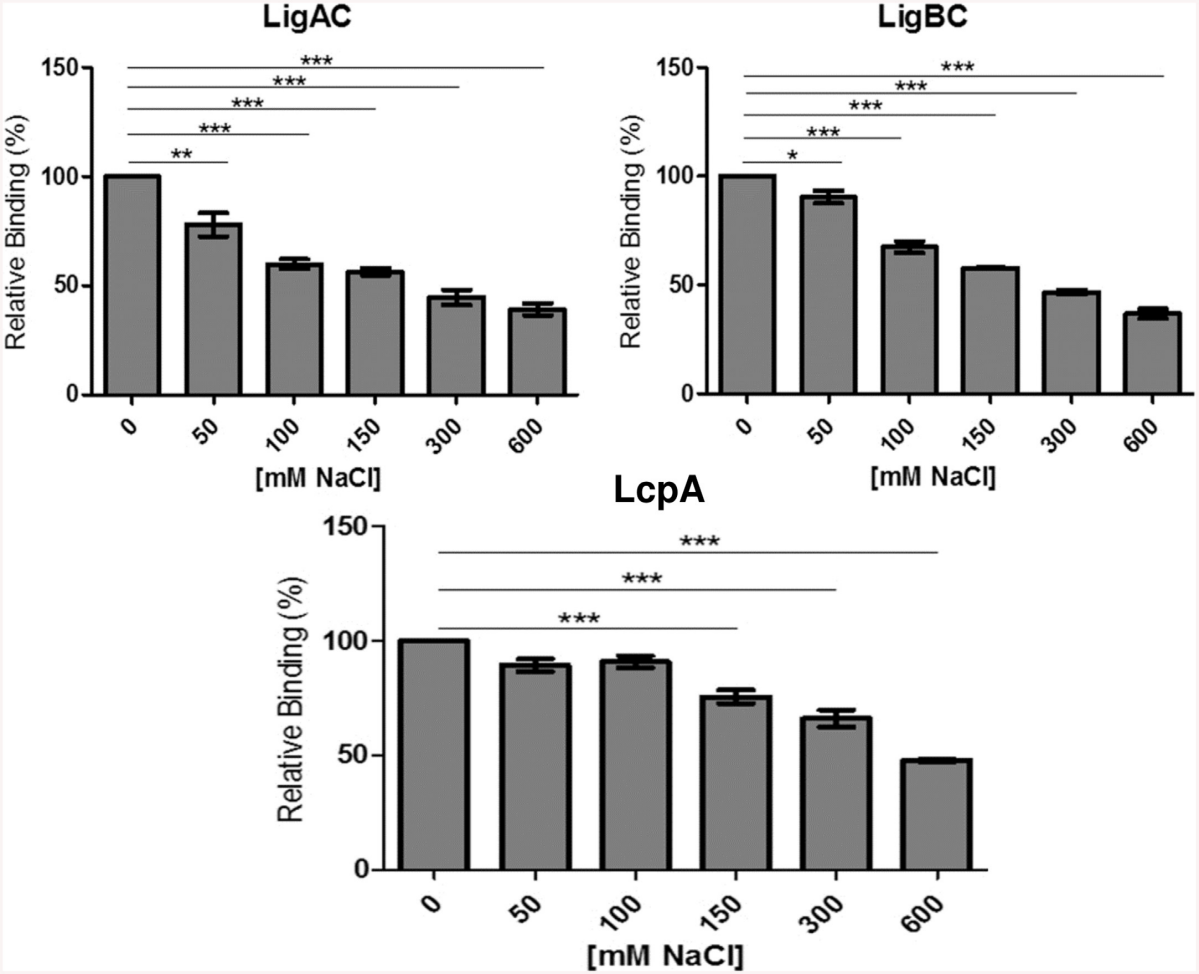
Interaction of C4BP and recombinant proteins of *L*. *interrogans* are dependent on ionic strength. LigA, LigB or LcpA were immobilized on microtiter wells. C4BP WT (commercial) diluted in 10 mM Na_2_HPO_4_ and 1.8mM KH_2_PO_4_ was prepared in different concentrations of NaCl and then added to the wells. To detect the binding of C4BP to recombinant proteins of *L*. *interrogans*, polyclonal rabbit anti-C4BP antibodies were used, followed by peroxidase-conjugated anti-rabbit IgG. Each point represents the mean absorbance value at 492 nm +/- the SD of 3 independent experiments, each performed in triplicate. The binding of recombinant proteins of *L*. *interrogans* and C4BP WT in the absence of NaCl was considered 100%. Data were analyzed using ANOVA test; *p<0.05; ***p<0.0001.

To further characterize the nature of the interaction of C4BP with LigA, LigB and LcpA, binding assays were performed using different concentrations of NaCl from 50 mM to 600 mM. As shown in Fig 6, the amount of LigAC and LigBC bound to C4BP is decreased by 45% at 150 mM NaCl while the amount of LcpA bound to C4BP decreased only 30% under these conditions. These results suggest that the interaction between C4BP and recombinant LcpA, LigAC and LigBC have a significant ionic component.

## Discussion

The complement system plays an important role in the innate and acquired immune responses, contributing to pathogen elimination. In 1955, Lawrence published one of the first studies about the role of complement activation in leptospiral infection, concluding that complement does not contribute to leptospiral killing [36]. However, in the 1960s, Johnsons published a series of three studies showing that complement plays an important role in leptospiral infection and demonstrated that saprophytic leptospires are more susceptible to lysis mediated by the complement system than pathogenic leptospires [37–39]. Since then, several groups worldwide have been interested in understanding the immune evasion mechanisms acquired by pathogenic strains of leptospires. In 2005, Meri and colleagues [40] showed that FH maintains its regulatory activity when bound to pathogenic leptospires, but does not bind to saprophytic leptospires. A variety of pathogens bind to complement inhibitors to evade complement system activation: relapsing fever spirochete *Borrelia* recurrentis binds to FH [41], C4BP [41] and C1 inhibitor [42] and *Neisseria gonorrhoeae* [43–44], *Streptococcus pyogenes* [45–46], *Haemophilus influenza* [47–48], *Moraxella catarrhalis* [49–50], *Bordetella pertussis* [51–52], *Escherichia coli* [53–54] and *Pasteurella pneumotropica* [55] interact with C4BP and FH. Not only bacteria, but also fungi have been shown to interact with C4BP, for example, *Candida albicans*[56] and *Aspergillus*[57]. In 2009, our group was the first to describe the interaction of C4BP with pathogenic leptospires and to show that C4BP bound to the leptospire surface is able to interact with and cleave C4b, regulating complement system activation [58].

**Fig 7:**
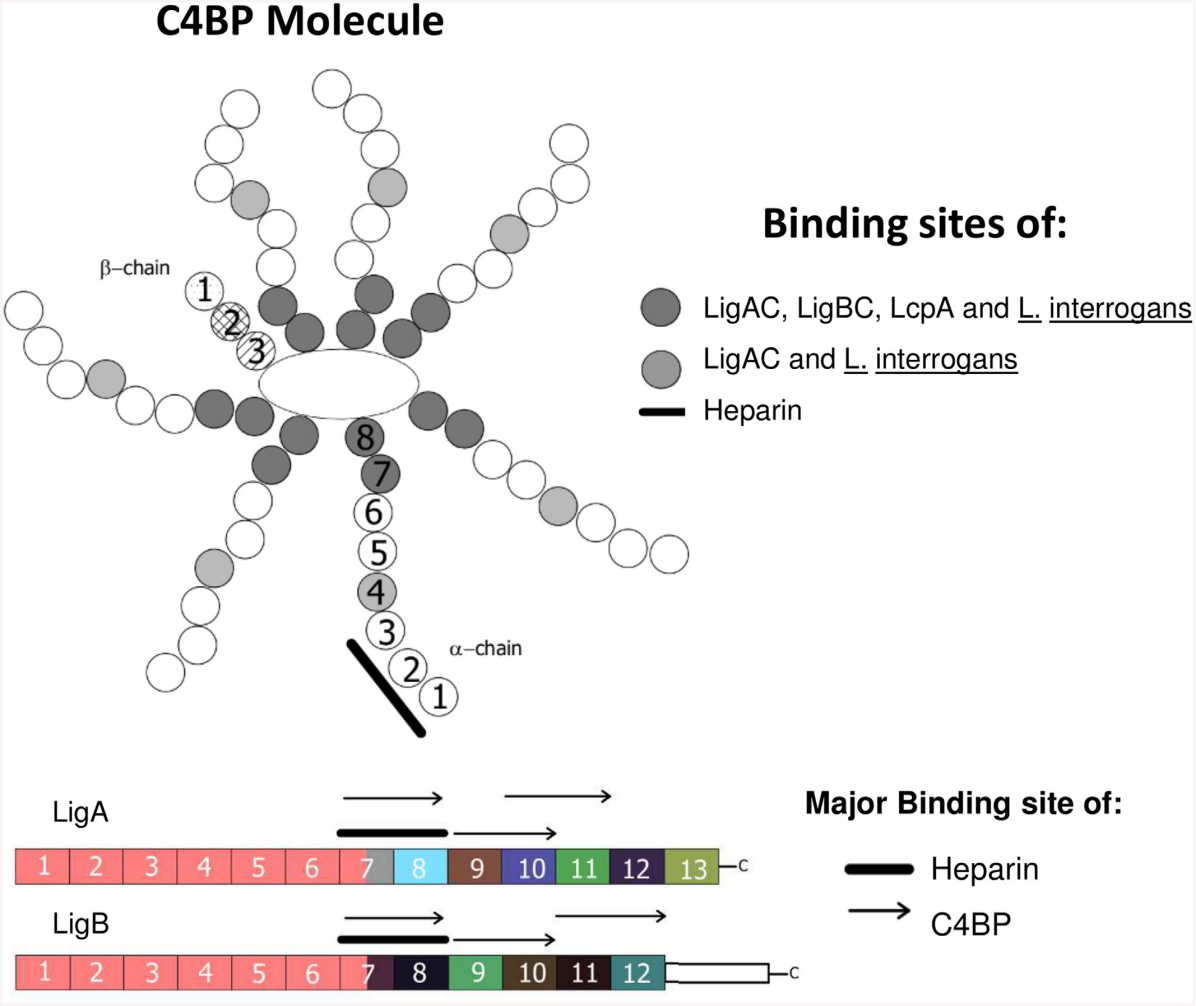
Summary of protein-protein and heparin-protein binding sites identified in this study. Schematic diagram of C4BP, Lig proteins and heparin binding sites. (**A**) LigAC, LigBC, LcpA and heparin binding sites on the C4BP molecule. C4BP CCP7 and CCP8 are binding sites for LigAC, LigBC, LcpA and whole *L*. *interrogans*. C4BP CCP4 is a binding site for only LigAC and whole *L*. *interrogans*. Heparin binds to the interface between CCP1 and CCP2 of C4BP [24]. (**B**) C4BP and heparin binding sites located on LigAC and LigBC proteins (numbers represent each Big domain). Note that C4BP and heparin compete for the same region on the C-terminus variable fragments of LigA and LigB molecules, respectively LigA7-8 and LigB7-8. C4BP also binds to LigA9-10, LigA10-11, LigB9-10 and LigB11-12.

LigA and LigB are multi-functional molecules closely associated with host infection and capable of inducing specific antibody responses [59–61]. We have previously identified three *L*. *interrogans* surface proteins that bind to C4BP: LcpA, LigA and LigB [15, 16]. In the present study, we mapped the binding sites of these proteins on the C4BP molecule using C4BP mutants lacking single CCPs of the α-chain. We observed that CCP4, CCP7 and CCP8 domains of C4BP α-chains are important for the interaction with LigAC and with intact *L*. *interrogans* whereas only CCP7 and CCP8 interact with purified LigBC and LcpA proteins, as summarized in Fig 7. Since the C4b binding site on C4BP is located on α-chain domains CCP1, CCP2 and CCP3 [28], C4BP bound to *Leptospira* probably retains cofactor activity. Such a strategy may help these spirochetes to regulate and inhibit the activation of the classical and lectin pathways inside the host. The same C4BP domains interact with *Streptococcus pnemoniae* [62], *Salmonella* [63], *Yersinia* [64], and *Haemophilus influenzae* [65]. Surprisingly, the interaction of C4BP with LigBC and with the leptospiral surface was increased in the presence of C4BP lacking CCP1 α-chain (Fig 2). One possible explanation is that C4BP lacking CCP1 may more easily adopt specific conformational states that yield tighter interactions with the leptospiral surface or LigBC. However, additional studies on the C4BP structure will be necessary to address this hypothesis. It is worth to mention that unexpected results have been also observed with *Porphyromonas gingivalis*, which display a stronger interaction with C4BP lacking CCP3 or CCP5 [66].

Next, we pinpointed the regions of LigA and LigB proteins capable of binding to C4BP. The conserved N-terminus region of LigA and LigB proteins does not interact significantly with C4BP compared to the C-terminus of LigA (LigAC) and LigB (LigBC) (Fig 3). Using a series of tandem Big domain constructs of Lig proteins, we observed that LigA7-8, LigA9-10 and LigA10-11 and LigB7-8, LigB9-10 and LigB11-12 are the main binding sites for C4BP on LigAC and LigBC, respectively. Several studies have used LigA and LigB constructs in order to map the regions of interaction with extracellular matrix components. Elastin and human tropoelastin bind to LigB7-8, LigB9 and LigB12 [11] while fibronectin binds to LigA7-8, LigA10, LigA11, LigA12, LigA13, LigB7-8, LigB9 and LigB12 domains [9, 67]. So, it seems that one ligand may have multiple binding sites on LigA and LigB proteins. Unlike elastin, human tropoelastin and fibronectin, C4BP is not able to bind to recombinant fragments containing a single Big domain derived from LigA or LigB proteins.

Although the binding sites of Lig proteins and heparin on the C4BP molecule do not overlap, binding of LigAC and LigBC to C4BP was inhibited by this highly sulphated glycosaminoglycan (Fig 4A) and we observed that both leptospiral proteins directly interact with heparin (Fig 4B). Using a series of tandem Big domain constructs of Lig proteins, we also observed that heparin binds preferentially to LigA7-8 and LigB7-8 (Fig 5A) and that heparin can inhibit the binding of LigA7-8, LigA10-11, LigB 7–8 and LigB9-10 fragments to C4BP (Fig 5B). Whether this inhibition is due to heparin binding to Lig proteins or to heparin binding to C4BP is not yet clear. However, since heparin´s interaction with C4BP may be stronger than its interaction with LigA and LigB we cannot rule out the possibility that heparin bound to C4BP CCPs 1–3 could interfere with or modify the binding of LigAC and LigBC to C4BP CCP4, 7 and 8 by way of an allosteric mechanism.

To date we still lack an efficient vaccine that protects people from different continents against leptospirosis. Lig proteins, notably LigA [13], have been shown to be the best vaccine candidates described so far. Unfortunately, they are not able to confer sterilizing immunity. A combination of different antigens, including Lig proteins, may eventually constitute an ideal vaccine against human leptospirosis. Understanding which regions of LigA and LigB are involved in the interactions with host´s complement regulators, such as C4BP, may contribute to the development of a human vaccine against the disease.
