# Correction: Fine Mapping of the Interaction between C4b-Binding Protein and Outer Membrane Proteins LigA and LigB of Pathogenic *Leptospira interrogans*

**DOI:** 10.1371/journal.pntd.0004286

**Published:** 2015-12-04

**Authors:** Leandro C. D. Breda, Ching-Lin Hsieh, Mónica M. Castiblanco Valencia, Ludmila B. da Silva, Angela S. Barbosa, Anna M. Blom, Yung-Fu Chang, Lourdes Isaac

The seventh author's name is spelled incorrectly. The correct name is: Yung-Fu Chang.
